# Partial Verification Bias Correction Using Inverse Probability Bootstrap Sampling for Binary Diagnostic Tests

**DOI:** 10.3390/diagnostics12112839

**Published:** 2022-11-17

**Authors:** Wan Nor Arifin, Umi Kalsom Yusof

**Affiliations:** 1 School of Computer Sciences, Universiti Sains Malaysia, Gelugor 11800, Pulau Pinang, Malaysia; 2 Biostatistics and Research Methodology Unit, School of Medical Sciences, Universiti Sains Malaysia, Kubang Kerian 16150, Kelantan, Malaysia

**Keywords:** correction method, diagnostic test, inverse probability bootstrap sampling, partial verification bias, propensity score

## Abstract

In medical care, it is important to evaluate any new diagnostic test in the form of diagnostic accuracy studies. These new tests are compared to gold standard tests, where the performance of binary diagnostic tests is usually measured by sensitivity (Sn) and specificity (Sp). However, these accuracy measures are often biased owing to selective verification of the patients, known as partial verification bias (PVB). Inverse probability bootstrap (IPB) sampling is a general method to correct sampling bias in model-based analysis and produces debiased data for analysis. However, its utility in PVB correction has not been investigated before. The objective of this study was to investigate IPB in the context of PVB correction under the missing-at-random assumption for binary diagnostic tests. IPB was adapted for PVB correction, and tested and compared with existing methods using simulated and clinical data sets. The results indicated that IPB is accurate for Sn and Sp estimation as it showed low bias. However, IPB was less precise than existing methods as indicated by the higher standard error (SE). Despite this issue, it is recommended to use IPB when subsequent analysis with full data analytic methods is expected. Further studies must be conducted to reduce the SE.

## 1. Introduction

Diagnostic tests play a central role in medical care; therefore, it is important to ensure the clinical validity of any new diagnostic tests [1,2] in the form of diagnostic accuracy studies. The validation involves comparing a new test with the clinically accepted gold standard test, where the performance of the new test is assessed by accuracy measures [1,3,4]. For binary diagnotic tests, sensitivity (Sn) and specificity (Sp) are commonly reported [3,4,5]. However, most often, the verification of disease status by the gold standard test is costly, time-consuming, and invasive [1,5,6,7,8]. This issue with verification causes partial verification bias (PVB), which occurs when only some patients are selected for disease verification by the gold standard test [1]. These patients are usually those with positive diagnostic test results, while those with negative test results are less likely to be selected [6,8,9]. Whenever the disease status is missing for some patients because it is not verified and the decision to verify depends on the result of the diagnostic test, this gives rise to the missing-at-random (MAR) missing data mechanism [5,6].

PVB is known to cause biased accuracy measures [1,6,10], so it is crucial to correct for this bias in analysis. Methods are available for PVB correction, depending on the scale of the diagnostic and gold standard tests, and the missing data mechanism. A recent review extensively covered all these methods [2], while a specific review on binary diagnostic and gold standard tests with practical implementation was covered in another article [11]. This study focused on PVB correction for binary diagnostic and gold standard tests under the MAR missing data mechanism.

For the binary diagnostic test and disease status (as verified by the gold standard test) under the MAR assumption, the available PVB correction methods can be roughly divided into Begg and Greene’s (BG)-based methods, propensity score (PS)-based methods, and multiple imputation (MI) method. BG-based and MI methods rely on estimating the probability of disease status given test result as an intermediate step before correcting the Sn and Sp estimates. This approach works because this probability, commonly known as positive and negative predictive values [3], is unbiased under MAR assumption [12]. PS-based methods estimate the probability of verification given the test result, before correcting for the bias by a weighting method [13,14]. By estimating the verification probability, PS demonstrates a clear and direct relationship with the verification problem, in this case, the PVB problem. Recent implementations of PVB correction methods can be seen in studies evaluating MRI and ultrasound in prostate cancer [15], serum pepsinogens in gastric cancer [16], and fine needle aspiration cytology in breast cancer [17], where the studies utilized BG-based and MI methods.

In a separate development in the field of ecology, Nahorniak et al. [18] proposed using inverse probability bootstrap (IPB) sampling in order to eliminate the effect of sampling bias in model-based analysis. Although bootstrap is generally known as a technique to obtain standard error of statistical estimates, they showed that the technique can also be utilized to obtain unbiased parameter estimates [18]. They achieved this by generating weighted bootstrap samples. IPB allows the use of the commonly used bootstrap technique that is easy to understand and apply by transforming the sample instead of having to modify or develop specific method to account for the bias [18]. Because IPB is basically a bootstrap technique, it allows easy estimation of the standard error of a parameter estimate to obtain the confidence interval for statistical inference, although it may require a cross-validation technique for this purpose in more complicated situations [18].

There is a common link between the PS-based method of PVB correction and IPB; both start with estimating the selection probability, or the verification probability in the context of PVB, before utilizing the probability to correct the bias by the same weighting methods. IPB offers an appealing approach to bias correction given its reliance on the bootstrap technique given the advantages of the technique. The weighted bootstrap sampling, as utilized by the IPB method, has not been investigated in the context of PVB correction, so its potential use and how it can be adapted in this context remains to be studied. Therefore, this study aimed to investigate the applicability of the IPB sampling method in the context of PVB correction under the MAR assumption for binary diagnostic tests.

## 2. Materials and Methods

This section describes the simulated and clinical data sets used in this study, the proposed implementation of IPB sampling for PVB correction, the metrics for performance evaluation, the selected methods for comparison, and the experimental setup of this study. In addition, the notations used are *T* = test result, *D* = disease status, and *V* = verification status.

### 2.1. Data Sets

Simulated and clinical data sets were used in this study for performance evaluation and comparison between the methods. The use of simulated data sets allow performance evaluation in comparison to known parameter values [18,19]. The use of real data sets allows for comparison between the methods using reference data sets, following the practice of previous research in PVB correction [20,21,22,23].

#### 2.1.1. Simulated Data Sets

The simulated data sets were generated by adapting the settings described in Harel and Zhou [21], Ünal and Burgut [22], and Rochani et al. [23]. The settings were as follows:

1. True disease prevalence (*p*) or P(D=1): moderate = 0.40 and low = 0.10.
2. True sensitivity (Sn) P(T=1|D=1): moderate = 0.6, high = 0.9.
3. True specificity (Sp) P(T=0|D=0): moderate = 0.6, high = 0.9.
4. Verification probabilities: When the verification depends only on test result, this gives an MAR missingness mechanism. Fixed verification probabilities given the test result P(V=1|T=t) were set at P(V=1|T=1) = 0.8 and P(V=1|T=0) = 0.4 [21]. In words, patients are more likely to be verified when their test results are positive with a probability of 0.8, while patients are less likely to be verified when their test results are negative with a probability of 0.4.
5. Sample sizes, *N*: 200 and 1000.

The probability of counts in the complete data of a 2×2 cross-tabulated table for test result *T* versus disease status *D*V=1) are distributed as a multinomial distribution [21,23]. Based on pre-specified Sn = P(T=1|D=1), Sp = P(T=0|D=0) and p=P(D=1)=π, the probabilities of counts are distributed as $M(\pi_{1},\pi_{2},\pi_{3},\pi_{4})$, where:

$$\begin{array}{cc} \pi_{1} & =P(T=1,D=1)=P(T=1|D=1)P(D=1),\\ \pi_{2} & =P(T=0,D=1)=P(T=0|D=1)P(D=1)=[1-P(T=1|D=1)]P(D=1),\\ \pi_{3} & =P(T=1,D=0)=P(T=1|D=0)P(D=0)=[1-P(T=0|D=0)]P(D=0),\\ \pi_{4} & =P(T=0,D=0)=P(T=0|D=0)P(D=0). \end{array}$$

Then, for each sample size setting *N*, the steps to generate a simulated PVB data set for MAR are as follows:

1. A complete data set of size *N* distributed as multinomial distribution, $M(\pi_{1},\pi_{2},\pi_{3},\pi_{4})$ was generated. This generated values ranging from 1 to 4 based on the probability values.
2. The values were converted into realizations of T=t and D=d variables, where 1→(T=1,D=1), 2→(T=0,D=1), 3→(T=1,D=0) and 4→(T=0,D=0).
3. Under the MAR assumption, a PVB data set with verification probability of P(V=1|T=1) = 0.8 and P(V=1|T=0) = 0.4 was generated.

#### 2.1.2. Clinical Data Sets

Two commonly used clinical data sets [21,23,24,25,26,27] to illustrate PVB correction methods were utilized. The original data in these studies were converted to an analysis-ready format (.csv). These data sets are described as follows:

1. Hepatic Scintigraphy Test
  The data set is about the hepatic scintigraphy test for the detection of liver cancer [24]. Hepatic scintigraphy is an imaging method (diagnostic test) to detect liver cancer. It was performed on 650 patients. Of the patients, 344 patients were later verified by liver pathological examination (gold standard test). The percentage of unverified patients is 47.1%. The data set contains the following variables:
  (a) Liver cancer, disease: Binary, 1 = Yes, 0 = No;
  (b) Hepatic Scintigraphy, test: Binary, 1 = Positive, 0 = Negative;
  (c) Verified, verified: Binary, 1 = Yes, 0 = No.
2. Diaphanography Test
  The data set is about the diaphanography test for detection of breast cancer [25]. Diaphanography is a noninvasive method (diagnostic test) of breast examination by transillumination using visible or infrared light to detect the presence of breast cancer. It was tested on 900 patients, where 88 patients were later verified by breast tissue biopsy for histological examination (gold standard test). The percentage of unverified patients is 90.2%. The data set contains the following variables:
  (a) Breast cancer, disease: Binary, 1 = Yes, 0 = No;
  (b) Diaphanography, test: Binary, 1 = Positive, 0 = Negative;
  (c) Verified, verified: Binary, 1 = Yes, 0 = No.

### 2.2. Inverse Probability Bootstrap Sampling

Nahorniak et al. [18] proposed inverse probability bootstrap (IPB) sampling to correct for selection bias, comprising of seven steps. In this study, the steps were adapted and simplified to five steps as follows:

1. Calculate selection probability $P_{i}$ from the biased sample of size *N* by any statistical method.
2. Calculate inverse sampling probability ($P_{i,IPB}$) as
  (1) $$P_{i,IPB}=\frac{\frac{1}{P_{i}}}{\sum_{i=1}^{n}\frac{1}{\sum_{i=1}^{n}P_{i}}},$$
  where $P_{i,IPB}$ is scaled such that the sum equals one and *n* is the sample size for complete cases.
3. Generate *b* bootsrap samples of size *n* by resampling with replacement *b* times.
4. Estimate parameter of interest as the mean of parameter estimates from the *b* bootstrap samples.
5. Estimate standard error (SE) as the standard deviation of the parameter estimates from the *b* bootstrap samples.

In this study, IPB sampling [18] was proposed for PVB correction by creating synthetic samples, where the samples are corrected for the bias. This was done by implementing Step (1) above using the propensity score ($PS_{i}$) in place of $P_{i}$, defined as

(2) $$PS_{i}=P\left(V_{i}=1|T_{i}\right),$$

where $PS_{i}$ may be known or is obtained from a logistic regression on the observed data [13,28,29]. Please note that in the context of PVB, the *n* specified in Step (2) (i.e., the size of complete cases after excluding observations with missing *D*) will be smaller than than the sample size in Step (1) (the size of full data containing *V* and *T* variables), denoted as *N*, where *n* equals *N* times the percentage of verification.

In Step (4), the parameters of interest are Sn and Sp. For each bootstrap sample, Sn and Sp estimates are calculated according the standard formula of Sn and Sp, as given in Equations (7) and (8), respectively. Following this calculation, the mean of the estimates for *b* bootstrap samples are calculated. In Step (5), the SE is utilized to obtain the 100(1−α)% confidence interval (CI) of the respective parameter estimate by bootstrap normal CI [30,31] as

(3) $$\hat{Sn}\pm z_{1-\alpha /2}\times SE_{bootstrap}\left(\hat{Sn}\right),$$

(4) $$\hat{Sp}\pm z_{1-\alpha /2}\times SE_{bootstrap}\left(\hat{Sp}\right).$$

Assuming the bootstrap distribution is approximately normal with small bias, the bootstrap normal interval gives a reasonable estimate [31]. Other common bootstrap intervals [30,31] are also possible as IPB is based on the bootstrap technique.

### 2.3. Performance Evaluation

#### 2.3.1. Performance Metrics

The performance evaluation was based on the metrics that measure the difference between an estimate and its true value [19,32,33]. The selected performance metrics, bias and standard error, are defined below. For a finite number of simulations *B*, these are calculated as follows:

1. Bias
  Bias of a point estimator $\hat{\theta }$ is the difference between the expected value of $\hat{\theta }$ and the true value of a parameter θ [33]. Bias is calculated as follows:
  (5) $$Bias=E\left[\hat{\theta }\right]-\theta =\frac{1}{B}\sum_{i=1}^{B}{\hat{\theta }}_{i}-\theta .$$
2. Standard Error
  Standard error (SE) is the square root of the variance, calculated as follows:
  (6) $$SE=\sqrt{Var(\hat{\theta })}=\sqrt{\frac{1}{B-1}\sum_{i=1}^{B}{\left({\hat{\theta }}_{i}-\bar{\theta }\right)}^{2}},$$
  where $\bar{\theta }$ is the mean of ${\hat{\theta }}_{i}$ across repetitions.

Bias is often the main metric of interest [19], where it indicates the accuracy of a method [33] and whether, on average, the method targets the parameter θ [19]. SE shows the precision of the method [19,33], where a smaller SE indicates better precision [33].

#### 2.3.2. Methods for Comparison

The following are existing methods for comparison with the IPB sampling method for PVB correction. Each method is described briefly, followed by the formula to calculate Sn and Sp. To compare the PVB correction methods, the BG method (for BG-based method), the inverse probability weighting estimator method (for PS-based method), and the MI method were selected to represent different approaches for PVB correction.

1. Full data analysis
  Full data analysis (FDA) represents the ideal analysis performed whenever full data are available without missing observations and bias, which is the standard way of calculating Sn and Sp. Sn and Sp for FDA [3] are calculated as follows:
  (7) $$\hat{Sn}{}_{FDA}=\hat{P}(T=1|D=1),$$
  (8) $$\hat{Sp}{}_{FDA}=\hat{P}(T=0|D=0).$$
2. Complete case analysis
  Complete case analysis (CCA) method accuracy estimates are calculated from the complete cases only [34]. CCA is biased in the presence of partial verification bias, and hence, represents the uncorrected method. Sn and Sp for CCA are calculated as follows:
  (9) $$\hat{Sn}{}_{CCA}=\hat{P}(T=1|D=1,V=1),$$
  (10) $$\hat{Sp}{}_{CCA}=\hat{P}(T=0|D=0,V=1).$$
3. Begg and Greenes’s method
  Begg and Greenes (BG) [35] proposed a correction method based on Bayes’ theorem whenever the missing data mechanism is MAR. Sn and Sp for the BG method [3,21,27,35] are calculated as follows:
  (11) $$\hat{Sn}{}_{BG}=\frac{\hat{P}\left(T=1\right)\hat{P}\left(D=1|T=1,V=1\right)}{\hat{P}\left(T=1\right)\hat{P}\left(D=1|T=1,V=1\right)+\hat{P}\left(T=0\right)\hat{P}\left(D=1|T=0,V=1\right)},$$
  (12) $$\hat{Sp}{}_{BG}=\frac{\hat{P}\left(T=0\right)\hat{P}\left(D=0|T=0,V=1\right)}{\hat{P}\left(T=1\right)\hat{P}\left(D=0|T=1,V=1\right)+\hat{P}\left(T=0\right)\hat{P}\left(D=0|T=0,V=1\right)}.$$
4. Inverse Probability Weighting Estimator
  Alonzo and Pepe [13] proposed the inverse probability weighting estimator (IPWE) method for PVB correction, which was based on the work of Horvitz and Thompson [36]. After estimating the verification probability $PS_{i}$, the IPWE method weights each observation in the verified sample by the inverse of the $PS_{i}$ to obtain the corrected Sn and Sp [13]. Sn and Sp for the IPWE method Alonzo and Pepe [13] are calculated as follows:
  (13) $${\hat{Sn}}_{IPWE}=\frac{\sum_{i=1}^{n}T_{i}V_{i}D_{i}/{\hat{PS}}_{i}}{\sum_{i=1}^{n}V_{i}D_{i}/{\hat{PS}}_{i}},$$
  (14) $${\hat{Sp}}_{IPWE}=\frac{\sum_{i=1}^{n}\left(1-T_{i}\right)V_{i}\left(1-D_{i}\right)/{\hat{PS}}_{i}}{\sum_{i=1}^{n}V_{i}\left(1-D_{i}\right)/{\hat{PS}}_{i}}.$$
5. Multiple Imputation
  Harel and Zhou [21] proposed using MI, where each missing disease status is replaced by m>1 plausible values, resulting in *m* complete data sets [5,21]. Each of these data sets is then analyzed by complete data methods; thereafter, the *m* estimates are combined to provide final estimates [5,21]. In this study, logistic regression was utilized in the imputation step of the MI method. The disease status was imputed using the following logistic regression model:
  (15) $$logit\left[P(D_{i}=1|T_{i})\right]=\beta_{0}+\beta_{1}\times T_{i},$$
  using the observed data. Following the imputation, Sn and Sp for the MI method are calculated as follows:
  (16) $${\bar{Sn}}_{MI}=\frac{1}{m}\sum_{j=1}^{m}{\hat{Sn}}_{FDA,j},$$
  (17) $${\bar{Sp}}_{MI}=\frac{1}{m}\sum_{j=1}^{m}{\hat{Sp}}_{FDA,j}.$$

For the simulated data sets, the methods are compared by the mean of the estimates and performance metrics (bias and SE), arranged by the sample sizes and Sn–Sp combinations. We did not consider *coverage*, i.e., the proportion of times the CI includes the estimate [19,32,33], as one of the performance metric for comparing the methods for the simulation. Because IPB is a bootstrap technique, the commonly used calculation to obtain the bootstrap CI was implemented and we did not propose a new method to obtain the CI.

For the clinical data sets, point estimates and the respective 95% CIs were estimated for comparison. For FDA and CCA, the CIs for Sn and Sp were calculated by using the Wald interval, while for the BG method, the calculation step given in the original article was followed [35]. For IPWE, the CIs were obtained by the bootstrap technique [13,14] using bootstrap bias-corrected and accelerated (BCa) interval [31]. For MI, the CIs were obtained by Rubin’s rule [11,37].

#### 2.3.3. Experimental Setup

R statistical programming language [38] version 3.6.3 was used to run the experiments within RStudio [39] integrated development environment. *mice* [40] version 3.14.0 and *simstudy* [41] version 0.5.0 R packages were used. The seed number for the random number generator was set to 3209673. Other experimental settings were the numbers of simulation runs *B* = 500 and bootstraps *b* = 1000 [22]. As for MI, the number of imputations was *m* = 100 for simulated data sets [42,43], while *m* = the percentage of incomplete cases for real clinical data sets [44,45,46].

## 3. Results

### 3.1. Simulated Data Sets

The simulation results for the FDA, CCA, and PVB correction methods are displayed in Table 1 for p=0.4. The results are arranged by the sample sizes N=200 and 1000, followed by Sn = (0.6, 0.9) and Sp = (0.6, 0.9) parameter combinations. The proportions of verification P(V=1) were 0.59, 0.52, and 0.64 for (0.6, 0.6), (0.6, 0.9), and (0.9, 0.6) for the (Sn, Sp) pairs, respectively. For experimental conditions with p=0.4, without any correction, using CCA for analysis resulted in biased estimates, while in the ideal research situation with the availability of full data, FDA showed very small bias. The results showed that for all PVB correction methods, including IPB, the bias values for Sn and Sp were very small for all Sn and Sp combinations, which reduced further at the larger N=1000. However, of all the correction methods, IPB displayed relatively larger SEs for both Sn and Sp estimation at N=200, while the SEs became smaller at N=1000.

Next, the simulation results for p=0.1 are displayed in Table 2. Similarly, the results are arranged by the sample sizes, followed by Sn and Sp parameter combinations. The proportions of verification were 0.57, 0.46, and 0.58 for (0.6, 0.6), (0.6, 0.9), and (0.9, 0.6) for (Sn, Sp) pairs, respectively. The results showed different patterns for p=0.1, where the bias values for Sp were very small for all Sn and Sp combinations at all sample sizes. All PVB correction methods, including IPB, showed small bias values of Sn for all Sn and Sp combinations. However, all correction methods underestimated the true Sn of 0.9, showing higher bias for the (Sn = 0.9, Sp = 0.6) combination at N=200, with MI having the highest bias value at −0.100, followed by −0.068 for IPB, and −0.063 for BG and IPWE. MI also showed relatively higher bias values of Sn for (Sn = 0.6, Sp = 0.6) and (Sn = 0.6, Sp = 0.9) combinations at N=200. CCA instead showed the smallest bias value even in comparison to FDA; this was because as CCA consistently overestimated Sn, at a higher Sn value, its estimate coincided with the true value, hence, the pseudo-good result. At N=1000, all PVB correction methods showed very small bias values of Sn. Similar to p=0.4, IPB displayed relatively larger SEs for both Sn and Sp estimation at N=200, although the SEs became smaller at N=1000.

### 3.2. Clinical Data Sets

The results for the PVB correction methods using the clinical data sets are displayed in Table 3. CCA is displayed to illustrate the results without bias correction. All correction methods, including the proposed IPB, showed closely similar point estimates for Sn and Sp of the hepatic data set and Sp of the diaphanography data set. The MI method showed slightly lower point estimate of Sn for the diaphanography data set as compared to the rest of the methods. For the hepatic data set, all methods showed relatively similar 95% CIs. For the diaphanography data set, the 95% CIs of Sn for BG and MI methods were close to each other, while the 95% CI of Sn for the IPB method was the widest of the rest of the methods. The same was observed for the 95% CI of Sp for the IPB method for this data set.

## 4. Discussion

The objective of this study was to investigate the applicability of IPB sampling method in the context of PVB correction. It was found that, based on the simulated data sets, the IPB method had good performance in terms of bias, although the SE was relatively larger than the other methods for comparison. Its performance was consistent for both moderate and low disease prevalence, while the MI method was the most affected at low disease prevalence. All methods showed better results at a larger sample size. All correction methods showed very small bias for Sp, while these methods varied in performance in correcting Sn estimates. Based on the clinical data sets, IPB was found to be consistent with other correction methods for the hepatic data set. For the diaphanogprahy data set, although the point estimates were consistent with other methods, the CIs were relatively wider than the rest of the methods.

Based on the results from the simulated data sets, in terms of bias, IPB was found to be as good as BG and IPWE in most experimental conditions, while being better than MI at low disease prevalence for estimating Sn. However, the SEs of Sn and Sp for IPB were larger than other methods, most notably at small sample size and low disease prevalence. As IPB only bootstraps the verified observations V=1, the bootstrapped sample size *n* is smaller than *N* (i.e., P(V=1)×N). This in effect leads to larger standard errors, as it is generally known that smaller sample sizes lead to larger standard errors [31]. This explains why, as *N* became larger, the SEs for IPB improved as *n* also became larger. In addition, IPB showed larger SEs in a low prevalence setting because the group size (D=1) became smaller with lower disease prevalence, where the size is p×n. Again, as *N* became larger, the SEs for IPB improved as the group size also became larger.

Next, based on the results from the clinical data sets, IPB showed consistent results for both the point and interval estimates for the hepatic data set. However, it showed wider 95% CIs of Sn and Sp based on the diaphanography data set. As observed in the simulated data sets, IPB exhibited a relatively large SE when the disease prevalence was low. The diaphanography data set has a large percentage of missing observations, which stood at 90.2%. Quite likely, the true disease prevalence was also low for this data set, although this could not be verified without the full data. At the same time, the observed sample size for the data set was only 88 patients. These factors might explain the wide CIs for IPB, while this also indicates IPB is sensitive to small sample sizes, which in effect will lead to larger SEs. As pointed out by Nahorniak et al. [18], although the SE for IPB was expected to be reasonably accurate, further study is required to study the performance of its SE.

While IPB was shown to be a viable alternative PVB correction method, its precision as indicated by the SE was slightly lower. Despite this shortcoming, there are several advantages of IPB over the other PVB correction methods. First, IPB was found to be less biased than MI at low disease prevalence, while being comparable to BG and IPWE in terms of bias. Second, IPB shares the same advantage with MI as both allows the use of any full data analytic methods, while BG and IPWE do not have the same advantage. The difference between IPB and MI was that, while MI restores full data *N* by imputing the missing values for the outcome *D*, IPB restores the correct distribution of the data containing the complete cases *n* only. The ability to utilize the full data approach is advantageous in applying new method for PVB correction as shown by Roldán-Nofuentes and Regad [47] in applying MI for the estimation of the average Kappa coefficient of a binary diagnostic test, where the IPB method might also be applicable. Third, it is straight forward to use IPB as opposed to MI, where it only requires the estimation of PS values, followed by the weighted bootstrap sampling procedure. In contrast, there are many imputation methods to choose from for MI [37], and the performance of MI depends on the chosen imputation method [48]. Finally, IPB shares the same advantage with IPWE by using PS (i.e., the probability of verification given test result, P(V=1|T=t)). While BG-based and MI methods rely on the correct probability of verification given test result, P(D=d|T=t), the PS-based methods rely on the correct P(V=1|T=t)) to perform the correction. Since P(D=d|T=t) will be incorrect when a case-control study design is used for diagnostic accuracy studies [8], the use of PS-based methods is advantageous in this situation.

## 5. Conclusions

PVB correction is important to ensure valid results for diagnostic accuracy studies affected by the PVB issue. Various correction methods have been developed to perform the correction, each with strengths and limitations. The IPB method is a general method to correct sampling bias in model-based analysis, and its utility in PVB correction has not been investigated before. This study investigated the IPB sampling method in the context of PVB correction under MAR assumption for binary diagnostic tests. The results showed that for PVB correction, IPB demonstrated low bias, indicating the method is accurate for estimation of Sn and Sp. However, IPB showed slightly higher SE than other correction methods that indicates the method is less precise. Despite this issue, as previously highlighted in the previous section, IPB has several advantages over other PVB correction methods. It is recommended to use IPB as an alternative to MI when debiased data are required for further analysis with full data analytic methods. Nevertheless, since the main disadvantage of IPB at this juncture is the larger SE, further research must be conducted to overcome this issue. In addition, since IPB by itself is a bootstrap technique, more research can be conducted on different bootstrap intervals to find the most suitable bootstrap interval in the context of PVB correction.

## Figures and Tables

**Table 1:** Comparison between IPB and existing PVB correction methods for *p* = 0.4 with N=200 and 1000 under three combinations of Sn and Sp.

Methods	Mean	Bias	SE	Mean	Bias	SE
	N=200					
	Sn = 0.6			Sp = 0.6		
FDA	0.603	0.003	0.055	0.602	0.002	0.044
CCA	0.754	0.154	0.060	0.430	−0.170	0.060
BG	0.607	0.007	0.072	0.602	0.002	0.050
IPWE	0.607	0.007	0.072	0.602	0.002	0.050
MI	0.605	0.005	0.075	0.599	−0.001	0.052
IPB	0.609	0.009	0.105	0.602	0.002	0.078
	Sn = 0.6			Sp = 0.9		
FDA	0.603	0.003	0.055	0.902	0.002	0.027
CCA	0.754	0.154	0.061	0.822	−0.078	0.054
BG	0.608	0.008	0.075	0.903	0.003	0.030
IPWE	0.608	0.008	0.075	0.903	0.003	0.030
MI	0.605	0.005	0.076	0.901	0.001	0.031
IPB	0.605	0.005	0.118	0.903	0.003	0.049
	Sn = 0.9			Sp = 0.6		
FDA	0.899	−0.001	0.033	0.601	0.001	0.044
CCA	0.945	0.045	0.027	0.427	−0.173	0.057
BG	0.896	−0.004	0.046	0.600	0.000	0.046
IPWE	0.896	−0.004	0.046	0.600	0.000	0.046
MI	0.889	−0.011	0.046	0.598	−0.002	0.047
IPB	0.894	−0.006	0.064	0.595	−0.005	0.072
	N=1000					
	Sn = 0.6			Sp = 0.6		
FDA	0.602	0.002	0.023	0.600	0.000	0.019
CCA	0.751	0.151	0.026	0.428	−0.172	0.026
BG	0.602	0.002	0.031	0.600	0.000	0.022
IPWE	0.602	0.002	0.031	0.600	0.000	0.022
MI	0.601	0.001	0.032	0.599	−0.001	0.022
IPB	0.599	−0.001	0.044	0.601	0.001	0.034
	Sn = 0.6			Sp = 0.9		
FDA	0.602	0.002	0.023	0.900	0.000	0.012
CCA	0.752	0.152	0.026	0.818	−0.082	0.025
BG	0.602	0.002	0.031	0.900	0.000	0.014
IPWE	0.602	0.002	0.031	0.900	0.000	0.014
MI	0.602	0.002	0.032	0.900	0.000	0.014
IPB	0.599	−0.001	0.048	0.899	−0.001	0.024
	Sn = 0.9			Sp = 0.6		
FDA	0.901	0.001	0.015	0.601	0.001	0.020
CCA	0.948	0.048	0.012	0.429	−0.171	0.027
BG	0.901	0.001	0.022	0.601	0.001	0.021
IPWE	0.901	0.001	0.022	0.601	0.001	0.021
MI	0.899	−0.001	0.023	0.600	0.000	0.021
IPB	0.901	0.001	0.028	0.600	0.000	0.033

Abbreviations: CCA, complete case analysis; BG, Begg and Greenes’ method; FDA, Full data analysis; IPWE, inverse probability weighting estimator; MI, multiple imputation; *N*, sample size; *p*, disease prevalence; SE, standard error; Sn, sensitivity; Sp, specificity; IPB, inverse probability bootstrap.

**Table 2:** Comparison between IPB and existing PVB correction methods for *p* = 0.1 with N=200 and 1000 under three combinations of Sn and Sp.

Methods	Mean	Bias	SE	Mean	Bias	SE
	N=200					
	Sn = 0.6			Sp = 0.6		
FDA	0.596	−0.004	0.112	0.601	0.001	0.037
CCA	0.743	0.143	0.117	0.429	−0.171	0.049
BG	0.603	0.003	0.144	0.601	0.001	0.038
IPWE	0.603	0.003	0.144	0.601	0.001	0.038
MI	0.579	−0.021	0.136	0.598	−0.002	0.039
IPB	0.595	−0.005	0.202	0.606	0.006	0.062
	Sn = 0.6			Sp = 0.9		
FDA	0.600	0.000	0.115	0.900	0.000	0.022
CCA	0.738	0.138	0.119	0.818	−0.082	0.043
BG	0.598	−0.002	0.143	0.900	0.000	0.023
IPWE	0.598	−0.002	0.143	0.900	0.000	0.023
MI	0.568	−0.032	0.134	0.900	0.000	0.023
IPB	0.599	−0.001	0.214	0.898	−0.002	0.042
	Sn = 0.9			Sp = 0.6		
FDA	0.875	−0.025	0.062	0.602	0.002	0.035
CCA	0.910	0.010	0.042	0.430	−0.170	0.048
BG	0.837	−0.063	0.068	0.599	−0.001	0.037
IPWE	0.837	−0.063	0.068	0.599	−0.001	0.037
MI	0.800	−0.100	0.080	0.597	−0.003	0.037
IPB	0.832	−0.068	0.133	0.605	0.005	0.059
	N=1000					
	Sn = 0.6			Sp = 0.6		
FDA	0.600	0.000	0.048	0.600	0.000	0.017
CCA	0.754	0.154	0.053	0.429	−0.171	0.022
BG	0.607	0.007	0.067	0.601	0.001	0.017
IPWE	0.607	0.007	0.067	0.601	0.001	0.017
MI	0.601	0.001	0.067	0.600	0.000	0.017
IPB	0.605	0.005	0.093	0.600	0.000	0.027
	Sn = 0.6			Sp = 0.9		
FDA	0.600	0.000	0.048	0.900	0.000	0.010
CCA	0.749	0.149	0.052	0.819	−0.081	0.019
BG	0.601	0.001	0.065	0.900	0.000	0.010
IPWE	0.601	0.001	0.065	0.900	0.000	0.010
MI	0.593	−0.007	0.065	0.900	0.000	0.010
IPB	0.604	0.004	0.099	0.901	0.001	0.018
	Sn = 0.9			Sp = 0.6		
FDA	0.899	−0.001	0.028	0.600	0.000	0.016
CCA	0.947	0.047	0.025	0.428	−0.172	0.021
BG	0.900	0.000	0.044	0.600	0.000	0.017
IPWE	0.900	0.000	0.044	0.600	0.000	0.017
MI	0.889	−0.011	0.046	0.600	0.000	0.017
IPB	0.897	−0.003	0.060	0.601	0.001	0.027

Abbreviations: CCA, complete case analysis; BG, Begg and Greenes’ method; FDA, Full data analysis; IPWE, inverse probability weighting estimator; MI, multiple imputation; *N*, sample size; *p*, disease prevalence; SE, standard error; Sn, sensitivity; Sp, specificity; IPB, inverse probability bootstrap.

**Table 3:** Sn and Sp estimates of IPB and other methods with the respective 95% CIs using clinical data sets.

	Hepatic Data Set		Diaphanography Data Set	
Methods	Sn (95% CI)	Sp (95% CI)	Sn (95% CI)	Sp (95% CI)
CCA	0.895 (0.858, 0.933)	0.628 (0.526, 0.730)	0.788 (0.648, 0.927)	0.800 (0.694, 0.906)
BG	0.836 (0.788, 0.884)	0.738 (0.662, 0.815)	0.292 (0.134, 0.449)	0.973 (0.958, 0.988)
IPWE	0.836 (0.784, 0.883)	0.738 (0.651, 0.809)	0.292 (0.165, 0.509)	0.973 (0.955, 0.986)
MI	0.834 (0.782, 0.885)	0.738 (0.661, 0.815)	0.279 (0.124, 0.435)	0.972 (0.957, 0.987)
IPB	0.838 (0.793, 0.882)	0.738 (0.653, 0.822)	0.290 (0.059, 0.520)	0.973 (0.935, 1.000)

Abbreviations: CCA, complete case analysis; CI, confidence interval; BG, Begg and Greenes’ method; FDA, Full data analysis; IPWE, inverse probability weighting estimator; MI, multiple imputation; Sn, sensitivity; Sp, specificity; IPB, inverse probability bootstrap.

## Data Availability

The data and code presented in this article are available from this GitHub repository: https://github.com/wnarifin/ipb_in_pvb (accessed on 10 November 2022).

## Abbreviations

The following abbreviations are used in this manuscript:

*b*	Number of bootstrap samples
*B*	Number of repetitions
BG	Begg and Greenes’ method
CCA	Complete case analysis
CI	Confidence interval
*D*	Disease status
FDA	Full data analaysis
IPB	Inverse probability bootstrap
IPWE	Inverse probability weighting estimator
*m*	Number of imputation
MAR	Missing at random
MI	Multiple imputation
*n*	Sample size for complete cases
*N*	Sample size
PVB	Partial verification bias
SE	Standard error
Sn	Sensitivity
Sp	Specificity
*T*	Test result
*V*	Verified
